# *Update*: Influenza Activity — United States and Worldwide, May 21–September 23, 2017

**DOI:** 10.15585/mmwr.mm6639a3

**Published:** 2017-10-06

**Authors:** Lenee Blanton, David E. Wentworth, Noreen Alabi, Eduardo Azziz-Baumgartner, John Barnes, Lynnette Brammer, Erin Burns, C. Todd Davis, Vivien G. Dugan, Alicia M. Fry, Rebecca Garten, Lisa A. Grohskopf, Larisa Gubareva, Krista Kniss, Stephen Lindstrom, Desiree Mustaquim, Sonja J. Olsen, Katherine Roguski, Calli Taylor, Susan Trock, Xiyan Xu, Jacqueline Katz, Daniel Jernigan

**Affiliations:** 1 Influenza Division, National Center for Immunization and Respiratory Diseases, CDC.

During May 21–September 23, 2017,* the United States experienced low-level seasonal influenza virus activity; however, beginning in early September, CDC received reports of a small number of localized influenza outbreaks caused by influenza A(H3N2) viruses. In addition to influenza A(H3N2) viruses, influenza A(H1N1)pdm09 and influenza B viruses were detected during May–September worldwide and in the United States. Influenza B viruses predominated in the United States from late May through late June, and influenza A viruses predominated beginning in early July. The majority of the influenza viruses collected and received from the United States and other countries during that time have been characterized genetically or antigenically as being similar to the 2017 Southern Hemisphere and 2017–18 Northern Hemisphere cell-grown vaccine reference viruses; however, a smaller proportion of the circulating A(H3N2) viruses showed similarity to the egg-grown A(H3N2) vaccine reference virus which represents the A(H3N2) viruses used for the majority of vaccine production in the United States. Also, during May 21–September 23, 2017, CDC confirmed a total of 33 influenza variant virus^†^ infections; two were influenza A(H1N2) variant (H1N2v) viruses (Ohio) and 31 were influenza A(H3N2) variant (H3N2v) viruses (Delaware [1], Maryland [13], North Dakota [1], Pennsylvania [1], and Ohio [15]). An additional 18 specimens from Maryland have tested presumptive positive for H3v and further analysis is being conducted at CDC.

## United States

**FIGURE 1:**
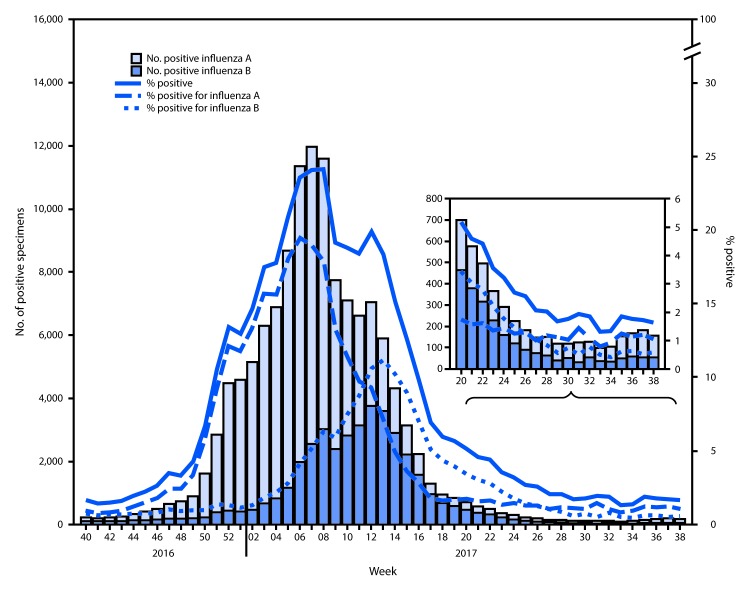
Number* and percentage of respiratory specimens testing positive for influenza reported by clinical laboratories, by influenza virus type and surveillance week — United States, October 2, 2016–September 23, 2017^†^ * 131,519 (12.3%) of 1,067,211 tested were positive during October 2, 2016–September 23, 2017. ^†^ As of September 29, 2017.

**FIGURE 2:**
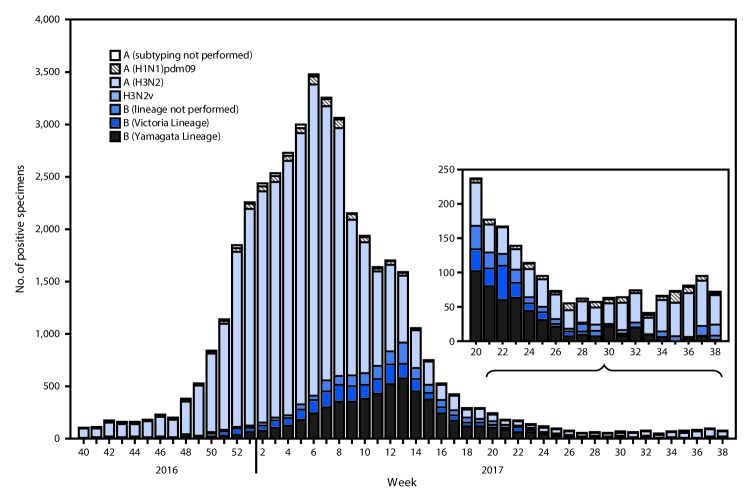
Number* of respiratory specimens testing positive for influenza reported by public health laboratories, by influenza virus type, subtype/lineage, and surveillance week — United States, October 2, 2016–September 23, 2017^†^ * N = 42,875. ^†^ As of September 29, 2017.

The U.S. Influenza Surveillance System^§^ is a collaboration between CDC and federal, state, local, and territorial partners and uses eight data sources to collect influenza information,^¶^ six of which operate year-round. U.S. World Health Organization (WHO) and National Respiratory and Enteric Virus Surveillance System laboratories, which include both public health and clinical laboratories throughout the United States, contribute to virologic surveillance for influenza. During May 21–September 23, 2017, clinical laboratories in the United States tested 153,397 respiratory specimens for influenza viruses, 3,785 (2.5%) of which were positive (Figure 1). Among these, 1,885 (49.8%) were positive for influenza A viruses, and 1,900 (50.2%) were positive for influenza B viruses. Public health laboratories in the United States tested 6,431 respiratory specimens collected during May 21–September 23, 2017. Among these, 1,536 were positive for influenza (Figure 2), including 842 (54.8%) that were positive for influenza A viruses, and 694 (45.2%) that were positive for influenza B viruses. Influenza B viruses were more commonly reported from late May through late June, and influenza A viruses have predominated since early July. Among the 828 (98.3%) influenza A viruses subtyped by public health laboratories, 715 (86.4%) were influenza A(H3N2) and 113 (13.6%) were influenza A(H1N1)pdm09 virus. Among the 537 (77.4%) influenza B viruses for which lineage was determined, 398 (74.1%) belonged to the B/Yamagata lineage and 139 (25.9%) belonged to the B/Victoria lineage.

During May 21–September 23, the weekly percentage of outpatient visits to health care providers for influenza-like illness** from the U.S. Outpatient Influenza-Like Illness Surveillance Network remained below the national baseline^††^ of 2.2%, ranging from 0.7% to 1.2%. Based on data from CDC’s National Center for Health Statistics Mortality Surveillance System, the percentage of deaths attributed to pneumonia and influenza did not exceed the epidemic threshold^§§^ and ranged from 5.1% to 6.1%. Four influenza-associated pediatric deaths occurring during May 21–September 23 were reported; two were associated with an influenza A(H3N2) virus, one was associated with an influenza A(H1N1)pdm09 virus, and one was associated with an influenza B virus.

## Novel Influenza A Virus Infections

Fifty-one human infections with novel influenza A viruses were reported in the United States during May 21–September 23, 2017. All of these were variant virus infections (human infections with influenza viruses that normally circulate in swine). Thirty-one have been sequenced and are H3N2v viruses reported from five states (Delaware [1], Maryland [13], North Dakota [1], Pennsylvania [1], and Ohio [15]) and two were H1N2v viruses, both from Ohio. The remaining 18 viruses have tested presumptive positive for H3v at the Maryland public health laboratory and further confirmatory testing is being performed by CDC. All 51 patients reported exposure to swine in a fair setting during the week preceding illness onset. Swine influenza A viruses were identified from respiratory specimens collected from pigs at multiple fairs. Forty-seven of the 51 patients were children aged <18 years and four patients were adults aged ≥50 years. Three of the 51 patients were hospitalized. All other patients are recovering or have fully recovered from their illness. No human-to-human transmission of these viruses has been identified.

The viruses detected in Maryland, Ohio, North Dakota, and Pennsylvania all had a hemagglutinin (HA) gene derived from a seasonal human H3N2 virus that was likely introduced into swine by reverse zoonosis (i.e., humans infecting swine) in 2010. These viruses were closely related to H3N2 viruses known to circulate in the U.S. swine population, as well as to variant virus infections detected in Ohio and Michigan during 2016 (*1*). Further analysis of the variant viruses detected in the most recent cases from Maryland is being performed at CDC. One of the H1N2v viruses had an HA gene from the alpha sublineage of the classical swine H1 HA lineage (*2*). This is the second alpha sublineage H1N2v virus detected since the mid-1990s. The second H1N2v virus had an HA gene representative of the delta 2 sublineage circulating in swine. The HA and neuraminidase (NA) genes are closely related to 2016/2017 swine influenza viruses from the United States. The NA genes of both H3N2v and H1N2v viruses are related to human H3N2 viruses that likely entered the North American swine population around 2002 and have remained the predominant NA found in contemporary swine influenza viruses.

## Worldwide

CDC serves as a WHO Collaborating Center for Surveillance, Epidemiology, and Control of Influenza, one of six WHO Collaborating Centers for Influenza in the WHO Global Influenza Surveillance and Response System (GISRS).^¶¶^ CDC, along with other international public health partners, provides surveillance and virus characterization data to WHO.*** The timing of influenza activity around the world varies by region,^†††^ and areas with similar influenza transmission patterns are grouped by influenza transmission zones.^§§§^

Reports from GISRS during May 21–September 23 suggested that typical seasonal patterns of influenza activity occurred in temperate climate Southern Hemisphere countries (*3*). Influenza activity began to increase in late April in the temperate countries of South America, late May in Southern Africa, early June in New Zealand, and early July in Australia. Influenza activity peaked in mid-June in temperate South America, the beginning of July in Southern Africa and New Zealand, and in mid-August in Australia, although elevated activity continued through September in Southern Africa and Australia. Influenza A(H3N2) viruses predominated across the Southern Hemisphere countries, with some reported influenza B viruses cocirculating in temperate South America, New Zealand, and Australia. In temperate-climate countries of Europe and North America, influenza activity was low, with influenza B viruses predominating.

In countries with tropical influenza seasonality, influenza activity levels and the predominant virus varied by country. In Central America and the Caribbean, activity was low and influenza A(H3N2) and influenza B viruses predominated. In tropical South America, influenza activity decreased from May 21 through September 23; influenza A(H3N2) viruses predominated with some influenza B viruses reported. Sporadic influenza virus detections were reported in Eastern and Western Africa, with influenza A(H1N1)pdm09, influenza A(H3N2), and influenza B viruses cocirculating. In Eastern Asia, high levels of influenza activity were reported in Southern China, Hong Kong special administrative region (SAR), and Taiwan beginning in July, peaked in mid-August, and decreased through September 23; influenza A(H3N2) viruses predominated. In Southern Asia, influenza A(H1N1)pdm09 viruses predominated, with elevated activity reported in India, Nepal, and the Maldives. Influenza activity in Southeast Asia was elevated in August and September. Influenza A(H1N1)pdm09 viruses predominated in the Philippines and Myanmar. Influenza A(H3N2), influenza A(H1N1)pdm09, and influenza B viruses cocirculated in Singapore, and influenza A(H1N1)pdm09 and influenza B viruses cocirculated in Vietnam.

During May 23–September 13, WHO reported 100 laboratory-confirmed human infections with avian influenza viruses, all from China, including 99 Asian lineage avian influenza A(H7N9) infections, and one influenza A(H9N2) infection.^¶¶¶^ A total of 764 human infections, including 283 (37%) deaths, with Asian lineage avian influenza A(H7N9) virus were reported to WHO from more provinces, regions, and municipalities in China during the fifth epidemic than in the previous four epidemics combined (*4*).

## Genetic and Antigenic Characterization of Influenza Viruses

The 2017–18 influenza vaccine virus components were selected in March 2017, during one of two biannual WHO-sponsored vaccine consultation meetings to review influenza data generated by GISRS laboratories. The recommended Northern Hemisphere 2017–18 trivalent influenza vaccine composition consists of an A/Michigan/45/2015 (H1N1)pdm09-like virus, an A/Hong Kong/4801/2014 (H3N2)-like virus, and a B/Brisbane/60/2008-like (B/Victoria lineage) virus. An additional influenza B virus (B/Phuket/3073/2013-like [B/Yamagata lineage]) was recommended for quadrivalent vaccines.**** These recommendations reflect an update to the A(H1N1)pdm09 virus component to a more contemporary influenza A(H1N1)pdm09 virus (an A/California/7/2009 (H1N1)pdm09-like virus was replaced with an A/Michigan/45/2015 (H1N1)pdm09-like virus), compared with the recommendation for the Northern Hemisphere 2016–2017 influenza season and are the same as the vaccine virus recommendations made for the 2017 Southern Hemisphere influenza vaccine.

Most influenza vaccines licensed in the United States, with the exception of cell culture–based inactivated influenza vaccine (ccIIV4) and recombinant influenza vaccines (RIV3 and RIV4) are produced through propagation of candidate vaccine viruses (CVVs) in eggs. Historically, CVVs provided to manufacturers have been egg-derived. Egg propagation of influenza viruses, particularly influenza A(H3N2) viruses, often leads to genetic changes that might have antigenic implications. The vaccine viruses selected for the Northern Hemisphere 2017–18 vaccine were representative of most, but not all, circulating influenza viruses at that time, and had the fewest and least substantial egg-adapted changes. In August 2016, the Food and Drug Administration approved the use of cell-derived CVVs for inclusion in ccIIV4.^††††^ For the 2017–18 season, the influenza A(H3N2) component of this vaccine is manufactured using a cell-derived CVV. The other components of this vaccine are manufactured using egg-derived CVVs. Production of influenza vaccines using cell-grown CVVs and cell-based technology can circumvent antigenic changes that might be associated with egg propagation, particularly for influenza A(H3N2) viruses.^§§§§^

Data obtained from antigenic characterization are important in the assessment of the similarity between reference vaccine viruses and circulating viruses. In vitro antigenic characterization data acquired through hemagglutination inhibition (HI) assays or virus neutralization assays are used to assess whether genetic changes in circulating viruses affect antigenicity, which could affect vaccine effectiveness. Since the 2014–15 season, many influenza A(H3N2) viruses lack sufficient hemagglutination titers for antigenic characterization using hemagglutination inhibition assays. Therefore, representative influenza A(H3N2) viruses are selected for antigenic characterization using the virus neutralization focus reduction assay to assess the ability of various antisera to neutralize infectivity of the test viruses. For nearly all influenza-positive surveillance samples received by CDC, next generation sequencing (NGS), which employs genomic enrichment practices (*5*–*7*), adapted by CDC, Nextera library preparation (Illumina, San Diego, California) and NGS using MiSeq (Illumina, San Diego, California), is performed to determine the genetic identity of circulating viruses. The genomic data are analyzed and submitted to public databases (GenBank or GISAID EpiFlu). CDC has antigenically or genetically characterized 877 influenza viruses collected and submitted by U.S. and international laboratories since May 21, 2017, including 117 influenza A(H1N1)pdm09 viruses, 495 influenza A(H3N2) viruses, and 265 influenza B viruses.

**FIGURE 3:**
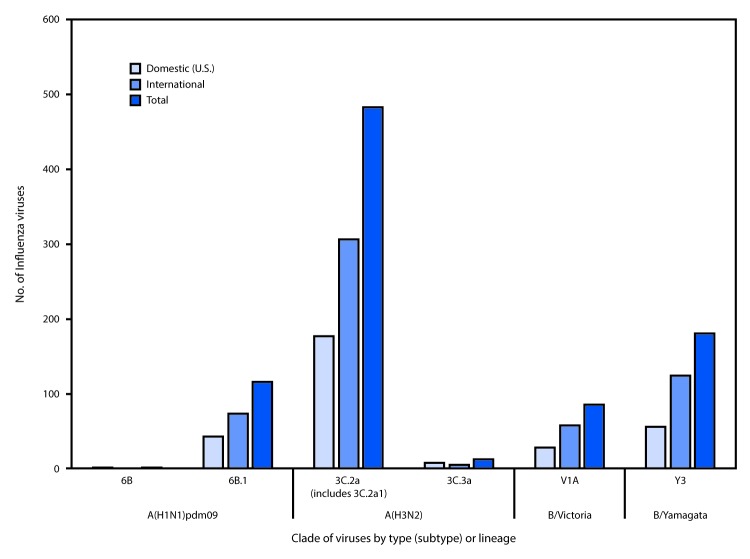
Genetic characterization of U.S. and international viruses collected during May 21, 2017–September 23, 2017* * As of September 29, 2017.

Phylogenetic analysis of the HA genes from the A(H1N1)pdm09 viruses collected since May 21, 2017, showed that all but one were in subclade 6B.1, and one virus belonged to clade 6B (Figure 3). All A(H1N1)pdm09 viruses were antigenically similar (analyzed using HI with ferret antisera) to the 6B.1 virus A/Michigan/45/2015, the recommended influenza A(H1N1)pdm09 reference virus for the 2017 Southern Hemisphere and 2017–18 Northern Hemisphere influenza vaccines.

Four hundred ninety-five influenza A(H3N2) viruses collected globally since May 21, 2017, were sequenced, and phylogenetic analysis of the HA genes illustrated that multiple clades/subclades were cocirculating (Figure 3). The HA genes showed extensive diversity and belonged to clades 3C.2a or 3C.3a, with 3C.2a predominating (Figure 3). The 3C.2a and the 3C.2a1 subclade circulated in approximately equal proportions. A representative set of 153 influenza A(H3N2) viruses (51 international and 102 United States) were antigenically characterized, and most (97%) A(H3N2) viruses were well-inhibited (reacting at titers of less than or equal to fourfold of the homologous virus titer) by ferret antisera raised against A/Michigan/15/2014 (3C.2a), a cell propagated A/Hong Kong/4801/2014-like reference virus representing the A(H3N2) component of the 2017 Southern Hemisphere and 2017–18 Northern Hemisphere influenza vaccines. A smaller proportion (33%) of influenza A(H3N2) viruses were well-inhibited by antiserum raised against egg-propagated A/Hong Kong/4801/2014 reference virus representing the A(H3N2) vaccine component, which is likely because of egg-adaptive amino acid changes in the HA of the egg-propagated virus.

A total of 85 influenza B/Victoria-lineage viruses were phylogenetically analyzed, and all HA genes belonged to genetic clade V1A, the same genetic clade as the vaccine reference virus, B/Brisbane/60/2008. However, two deletion subclades were detected in 2017. One subclade has a 6-nucleotide deletion (encoding amino acids 162 and 163) and the other subclade has a 9-nucleotide deletion (encoding amino acids 162, 163 and 164). The 162–163 double deletion in the HA was detected in viruses circulating in multiple countries, with the majority identified in the United States, although the three viruses with 162–164 triple deletion were only detected in Hong Kong SAR, China. Thirty-nine (72%) B/Victoria lineage viruses were well-inhibited by ferret antisera raised against MDCK-propagated B/Brisbane/60/2008 reference virus, representing the B/Victoria lineage component of the 2017 Southern Hemisphere and 2017–2018 Northern Hemisphere influenza vaccines. However, 28% of B/Victoria lineage viruses reacted poorly with ferret antisera raised against MDCK-propagated B/Brisbane/60/2008, which correlated with the 162–163 double deletion and the 162–164 triple deletion in the HA.

Phylogenetic analysis of 180 influenza B/Yamagata-lineage viruses indicate that the HA genes belonged to clade Y3 (Figure 3). A total of 99 representative influenza B/Yamagata-lineage viruses (59 international and 40 United States) were antigenically characterized, and all were antigenically similar to B/Phuket/3073/2013, the reference vaccine virus representing the influenza B/Yamagata-lineage component of the 2017 Southern Hemisphere and 2017–18 Northern Hemisphere quadrivalent vaccines.

## Composition of the 2018 Southern Hemisphere Influenza Vaccine

The WHO recommendations for influenza vaccine composition for the 2018 Southern Hemisphere season were made at the WHO Vaccine Consultation meeting September 25–28, 2017, in Melbourne, Australia. The recommended components for the 2018 Southern Hemisphere influenza trivalent vaccines are an A/Michigan/45/2015 (H1N1)pdm09-like virus, an A/Singapore/INFIMH-16-0019/2016 (H3N2)-like virus, and a B/Phuket/3073/2013-like (B/Yamagata lineage) virus (*8*). For quadrivalent vaccines, an additional component, B/Brisbane/60/2008-like (B/Victoria lineage) virus, is recommended (*8*). This represents a change in the influenza A(H3N2) component and a change in the influenza B lineage included in the trivalent vaccine compared with the composition of the 2017 Southern Hemisphere and 2017–18 Northern Hemisphere influenza vaccine formulation. The H3N2 component was updated to address the egg-adaptive changes that occurred with the egg-propagated A/Hong Kong/4801/2014 reference virus and to better represent genetic changes seen in recently circulating H3N2 viruses.

## Antiviral Resistance of Influenza Viruses

The WHO Collaborating Center for Surveillance, Epidemiology, and Control of Influenza at CDC tested 486 influenza virus specimens collected during May 21–September 23, 2017, from the United States and worldwide for resistance to the influenza neuraminidase inhibitor antiviral medications currently approved for use against seasonal influenza: oseltamivir, zanamivir, and peramivir. A total of 75 influenza A(H1N1)pdm09 viruses (37 international and 38 United States) were tested, and all were sensitive to these drugs. All 231 influenza A(H3N2) viruses (60 international and 171 United States) and all 180 influenza B viruses (98 international and 82 United States) tested were also sensitive to all three recommended antiviral medications. High levels of resistance to the adamantanes (amantadine and rimantadine) persist among influenza A(H1N1)pdm09 and influenza A(H3N2) viruses. Adamantane drugs continue not to be recommended for use against influenza at this time.

## Discussion

During May 21–September 23, 2017, influenza A(H3N2), influenza A(H1N1)pdm09, and influenza B viruses co-circulated worldwide. In the United States, influenza B viruses predominated from late May through late June. Influenza A viruses were most commonly reported beginning in early July. The majority of the influenza viruses collected from the United States and other countries during that time were characterized antigenically and genetically as being similar to the cell-grown reference viruses representing the 2017 Southern Hemisphere and 2017–18 Northern Hemisphere influenza vaccine viruses. Antigenic and genetic characterization of circulating influenza viruses can give an indication of the influenza vaccine's ability to produce an immune response against the wide array of influenza viruses cocirculating, but vaccine effectiveness studies are needed to determine how much protection has been provided to the population by vaccination. Influenza A(H1N1)pdm09 viruses were detected at low levels from May 21 to September 23, and virus characterization data indicate no substantial genetic or antigenic changes, even among viruses from regions that experienced higher A(H1N1)pdm09 activity. Influenza A(H3N2) viruses have predominated in many countries in the Southern Hemisphere and in the United States since early July. Virus characterization data suggest extensive genetic diversity among circulating viruses, but limited evidence of substantial antigenic drift. To date, a predominant subclade of A(H3N2) viruses with substantial antigenic drift has yet to emerge, and extensive genetic variation exists in the circulating virus population. Among the influenza B viruses for which lineage was determined, influenza B/Yamagata viruses predominated across the United States from May 21 through September 23, and virus characterization data indicate no substantial genetic or antigenic changes. Two subgroups of antigenically distinct influenza B/Victoria viruses, represented by the double or triple deletion viruses, were detected; the majority of the double deletion viruses were identified in the United States, while all three triple deletion viruses were identified only in Hong Kong SAR, China. Nevertheless, such antigenically distinct viruses represented a minority of B/Victoria viruses circulating globally during this period. Close monitoring of these viruses is required to better assess their potential impact on public health. Although influenza B viruses circulate throughout the influenza season, they frequently circulate later in the season than do influenza A viruses and often result in a second peak of influenza activity, often in the late winter and spring in the United States and other Northern Hemisphere countries (*3*).

Annual influenza vaccination is the best method for preventing influenza and its potentially severe complications (*9*). In the United States, annual influenza vaccination is recommended for all persons aged ≥6 months who do not have contraindications. Annual influenza vaccination is recommended regardless of whether the vaccine composition has changed because immunity from vaccination wanes over time and might decline below protective levels after one season. Optimally, vaccination should occur before the onset of influenza activity in the community. If possible, vaccination should be offered by the end of October and should continue to be offered as long as influenza viruses are circulating and unexpired vaccine is available. Children aged 6 months through 8 years who require 2 doses should receive their first dose as soon as possible after vaccine becomes available, and the second dose ≥4 weeks later. For 2017-18 season, manufacturers have projected they will supply the United States with as many as 151 to 166 million doses of injectable influenza vaccine; approximately 119 million of this will be quadrivalent vaccine. As of September 15, 2017, approximately 73 million doses had already been distributed.

Multiple influenza vaccines are approved and recommended for use and are being distributed during the 2017–18 season, including egg-based trivalent and quadrivalent inactivated influenza vaccines (IIV3 and IIV4), adjuvanted trivalent egg-based inactivated influenza vaccines (aIIV3), high-dose trivalent egg-based inactivated influenza vaccines (HD-IIV3), quadrivalent cell culture–based inactivated influenza vaccines (ccIIV4), and recombinant trivalent and quadrivalent influenza vaccines (RIV3 and RIV4). Two available intramuscular vaccines are approved for administration by jet injector for persons aged 18 through 64 years. One IIV4 formulation is approved for intradermal administration. There is no preferential recommendation for one licensed and recommended influenza vaccine product over another for persons for whom more than one licensed, recommended product is available (*9*). Currently available influenza vaccines, with the exceptions of RIV3, RIV4, and ccIIV4, are prepared by propagation of virus in embryonated eggs (*9*). Egg propagation of influenza A(H3N2) viruses often leads to genetic changes that have antigenic implications. For the 2017–18 season inactivated vaccines, all influenza A(H1N1) and A(H3N2) and both influenza B components will be egg-derived, with the exception of ccIIV4, for which the influenza A(H3N2) virus component will, for the first time, be a cell-derived vaccine virus component (*9*). This represents a first step toward producing a totally egg-independent inactivated virus vaccine. Recombinant technology is used in the production of RIV3 and RIV4; therefore they are manufactured without the use of influenza viruses or eggs. The use of egg-independent vaccine technologies is likely to provide vaccines that more precisely represent the antigenic characteristics of circulating viruses and have the potential to offer improved protection. Because of the low effectiveness of live attenuated intranasal influenza vaccine (LAIV4) against influenza A(H1N1)pdm09 viruses in the United States during the 2013–14 and 2015–16 seasons, for the 2017–18 season, the Advisory Committee on Immunization Practices and CDC renewed the recommendation that LAIV4 should not be used (*9*).

Although vaccination is the best method for preventing and reducing the impact of influenza, antiviral medications provide a valuable adjunct. Treatment with influenza antiviral medications as early as possible in the course of illness is recommended for patients with confirmed or suspected influenza (either seasonal influenza or novel influenza virus infection) who have severe, complicated, or progressive illness; who require hospitalization; or who are at high risk for influenza-related complications^¶¶¶¶^ (*10*). Treatment is most effective when given early in the illness, especially within 48 hours of illness onset; providers should not delay treatment until test results become available and should not rely on insensitive assays such as found with some rapid antigen detection influenza diagnostic tests to determine treatment decisions (*10*).

Fifty-one infections with variant influenza viruses were reported from five states during summer 2017. Most of these infections occurred in children with prior direct contact with pigs at agricultural fairs, highlighting the importance of preventive actions,***** especially for young children or persons at high risk for serious influenza complications. Although community transmission of these viruses has not been identified, the potential for them to develop the ability to transmit efficiently from person to person remains a concern. Testing for seasonal influenza viruses and monitoring for novel influenza A virus infections should continue year-round. Health care providers also are reminded to consider novel influenza virus infections in persons with influenza-like illness and swine or poultry exposure, or with severe acute respiratory infection after travel to areas where avian influenza viruses have been detected. Providers should alert the local public health department if novel influenza virus infection is suspected. Clinical laboratories using a commercially available influenza diagnostic assay that includes influenza A virus subtype determination should contact their state public health laboratory to facilitate transport and additional testing of any specimen that is positive for influenza A, but for which the subtype cannot be determined. Public health laboratories should immediately send influenza A virus specimens that they cannot subtype using standard methods to CDC and submit all specimens that are otherwise unusual as soon as possible after identification. Early identification and investigation of human infections with novel influenza A viruses are critical to ensure timely risk assessment so that appropriate public health measures can be taken.

Influenza surveillance reports for the United States are posted online weekly (https://www.cdc.gov/flu/weekly). Additional information regarding influenza viruses, influenza surveillance, influenza vaccine, influenza antiviral medications, and novel influenza A infections in humans is available at https://www.cdc.gov/flu.

**Summary**

**What is already known about this topic?**

CDC collects, compiles, and analyzes data on influenza activity year-round in the United States. Timing of influenza activity and predominant circulating influenza viruses vary by season.

**What is added by this report?**

Worldwide, influenza activity during May 21–September 23, 2017, followed typical seasonality and in the United States overall, low levels of seasonal influenza activity were detected. The majority of influenza viruses genetically and antigenically characterized at CDC were similar to the reference viruses representing the recommended components for the 2017–18 vaccine. A small subset of antigenically distinct influenza B/Victoria viruses was detected.

**What are the implications for public health practice?**

In the United States, an annual influenza vaccination is recommended for all persons aged ≥6 months and can reduce the likelihood of becoming ill with influenza and transmitting the virus to others. Annual influenza vaccination offers optimal protection regardless of whether the vaccine composition has changed since the previous season, because immunity wanes over time. Although vaccination is the best method for preventing and reducing the impact of influenza, antiviral medications are an important adjunct. Early treatment with influenza antiviral medications is recommended for patients with confirmed or suspected influenza (either seasonal influenza or novel influenza virus infection) who have severe, complicated, or progressive illness; who require hospitalization; or who are at high risk for influenza-related complications. Testing for seasonal influenza viruses and monitoring for novel influenza A virus infections should continue year-round.

## Footnotes

* Data as of September 29, 2017.

† Influenza viruses that circulate in swine are called swine influenza viruses when isolated from swine, but are called variant influenza viruses when isolated from humans. Seasonal influenza viruses that circulate worldwide in the human population have important antigenic and genetic differences from influenza viruses circulating in swine.

§ The CDC influenza surveillance system collects five categories of information from eight data sources: 1) viral surveillance (U.S. World Health Organization collaborating laboratories, the National Respiratory and Enteric Virus Surveillance System, and novel influenza A virus case reporting); 2) outpatient illness surveillance (U.S. Outpatient Influenza-Like Illness Surveillance Network); 3) mortality (the National Center for Health Statistics Mortality Surveillance System and influenza-associated pediatric mortality reports); 4) hospitalizations (FluSurv-NET, which includes the Emerging Infections Program and surveillance in three additional states); and 5) summary of the geographic spread of influenza (state and territorial epidemiologist reports). https://www.cdc.gov/flu/weekly/fluactivitysurv.htm.

¶
https://www.cdc.gov/flu/weekly/overview.htm .

** Defined as a fever (temperature ≥100°F [≥37.8°C]), oral or equivalent, and cough and/or sore throat, without a known cause other than influenza.

†† The national and regional baselines are the mean percentage of visits for influenza-like illness (ILI) during noninfluenza weeks for the previous three seasons plus two standard deviations. Noninfluenza weeks are defined as periods of ≥2 consecutive weeks in which each week accounted for <2% of the season’s total number of specimens that tested positive for influenza in public health laboratories. National and regional percentages of patient visits for ILI are weighted based on state population. Use of the national baseline for regional data is not appropriate.

§§ The seasonal baseline proportion of pneumonia and influenza (P&I) deaths is projected using a robust regression procedure, in which a periodic regression model is applied to the observed percentage of deaths from P&I that were reported by the National Center for Health Statistics Mortality Surveillance System during the preceding 5 years. The epidemic threshold is set at 1.645 standard deviations above the seasonal baseline.

¶¶
http://www.who.int/influenza/gisrs_laboratory/collaborating_centres/en .

***
http://www.who.int/influenza/gisrs_laboratory/en/ .

††† In temperate climates, the onset and peak of influenza activity might vary substantially from one influenza season to the next, but generally begins to increase in the late fall. In the Northern Hemisphere's temperate regions, annual epidemics of influenza typically occur during October–February, but the peak of influenza activity can occur as late as April or May. In temperate regions of the Southern Hemisphere, influenza activity typically peaks during May through August. Although temperate regions of the world experience a seasonal peak in influenza activity, influenza viruses can be isolated year-round. The timing of seasonal peaks in influenza activity in tropical and subtropical countries varies by region. Multiple peaks of activity during the same year have been seen in some areas and influenza infection can occur year-round.

§§§
http://www.who.int/influenza/gisrs_laboratory/flunet/en/ .

¶¶¶ The list of WHO monthly risk assessment summaries for human infections with avian influenza viruses is available at http://www.who.int/influenza/human_animal_interface/HAI_Risk_Assessment/en/ and WHO disease outbreak news reports are available at http://www.who.int/csr/don/en/.

****
http://www.who.int/influenza/vaccines/virus/recommendations/2017_18_north/en/ .

†††† https://www.fda.gov/downloads/BiologicsBloodVaccines/Vaccines/ApprovedProducts/UCM522280.pdf.

§§§§ https://virologyj.biomedcentral.com/track/pdf/10.1186/1743-422X-4-42?site=virologyj.biomedcentral.com.

¶¶¶¶ Persons at high risk include 1) children aged <2 years; 2) adults aged ≥65 years; 3) persons with chronic pulmonary conditions (including asthma), cardiovascular disease (except hypertension alone), renal, hepatic, hematologic (including sickle cell) disease, metabolic disorders (including diabetes mellitus), or neurologic and neurodevelopmental conditions (including disorders of the brain, spinal cord, peripheral nerves, and muscles, such as cerebral palsy, epilepsy [seizure disorders], stroke, intellectual disability [mental retardation], moderate to severe developmental delay, muscular dystrophy, or spinal cord injury); 4) persons with immunosuppression, including that caused by medications or by human immunodeficiency virus infection; 5) women who are pregnant or postpartum (within 2 weeks after delivery); 6) persons aged ≤18 years who are receiving long-term aspirin therapy; 7) American Indians/Alaska Natives; 8) persons with extreme obesity (i.e., body mass index ≥40); and 9) residents of nursing homes and other chronic care facilities.

***** https://www.cdc.gov/flu/swineflu/variant/preventspreadfactsheet.htm.
